# Parental Feeding, Child Eating and Physical Activity: Differences in Children Living with and without Asthma

**DOI:** 10.3390/ijerph18073452

**Published:** 2021-03-26

**Authors:** Rebecca Clarke, Gemma Heath, Prasad Nagakumar, Helen Pattison, Claire Farrow

**Affiliations:** 1 School of Psychology, Aston University, Birmingham B4 7ET, UK; g.heath1@aston.ac.uk (G.H.); h.m.pattison@aston.ac.uk (H.P.); c.farrow@aston.ac.uk (C.F.); 2 Department of Paediatric Respiratory Medicine and Cystic Fibrosis, Birmingham Women’s and Children’s Hospital, Birmingham B4 6NH, UK; p.nagakumar@nhs.net

**Keywords:** asthma, parents, adolescence, weight management, feeding, exercise

## Abstract

This study aimed to establish the differences in parental attitudes toward feeding and activity, as well as child eating and activity levels, between families of children living with and without asthma. Parents of children and young people aged between 10 and 16 years living both with asthma (n = 310) and without asthma (n = 311) completed measures for parental feeding, parental attitudes toward child exercise, child eating, child activity level and asthma control. Children living with asthma had a significantly higher BMIz (BMI standardised for weight and age) score, were significantly more likely to emotionally overeat and desired to drink more than their peers without asthma. Parents of children with asthma reported greater use of food to regulate emotions, restriction of food for weight control, monitoring of child activity, pressure to exercise and control over child activity. When asthma symptoms were controlled, parental restriction of food for weight management predicted greater child BMIz scores, and higher child activity predicted lower child BMIz scores. These relationships were not found to be significant for children with inadequately controlled asthma. Differences in parental attitudes toward feeding and exercise, and child eating and exercise behaviors, between families may help to explain the increased obesity risk for children with asthma.

## 1. Introduction

Asthma is one of the most common chronic illnesses in children and young people [1]. The exacerbation of asthma symptoms can impose a significant burden upon the family and society, accounting for missed school and workdays, hospitalizations and a decreased quality of life [2,3,4,5]. When young people are also living with overweight/obesity, the prevalence and severity of asthma symptoms is understood to increase [6]. Longer hospital stays and reduced effectiveness of inhaled corticosteroids among this group highlight the increased challenges to asthma management [7,8]. Emerging evidence suggests that weight management in this population can help to reduce asthma symptom severity [9]. To inform the development of future asthma-specific weight management interventions, a greater understanding is needed of modifiable factors that influence health behaviors in asthma management. 

Health behaviors are often established in early childhood. Evidence suggests that parental feeding practices influence a child’s own eating behaviors that are maintained going into and throughout adulthood [10,11]. Parents of children living with chronic illness may have different approaches to feeding. Greater parental use of food to counter-act negative emotions, greater restriction of food and more monitoring of dietary intake have been reported in parents of children living with chronic illness compared to parents of children without chronic illnesses [12,13,14]. Despite research suggesting that a healthy diet correlates with reduced asthma symptoms, there is limited understanding of how parental feeding and child eating may differ in those with pediatric asthma [15]. Borhani and colleagues [16] found that parents of children living with asthma reported hypervigilant supervision of their child’s dietary intake. Moreover, population-based cohort studies have observed increased unhealthy eating behaviors in adolescents with asthma [17]. 

Living with asthma also poses challenges for physical activity. Exercise can trigger asthma symptoms, resulting in wheezing, shortness of breath and coughing during or after physical activity. Difficulty caused by exercise-induced bronchoconstriction (EIB) during physical activity may explain why pediatric asthma is associated with reduced participation in physical activity and a higher child BMI [18]. However, if therapeutic treatments for asthma fail to prevent EIB, other causes of breathlessness such as physiologic limitations should be considered [19]. Park, Sawyer and Glaun [20] propose that breathlessness during activity could be misinterpreted as asthma exacerbation in some families. This may be a factor in determining how much exercise families and children living with asthma engage in [21,22]. Thus, parental fears of EIB may create barriers to physical activity which are unintentionally transferred to the child [21,23]. Such barriers are problematic for weight and illness management, with research indicating that exercise can increase lung function and cardio-pulmonary fitness and improve asthma control [2]. 

The adjustment of health behaviors, such as eating and exercise, as a management strategy to avoid asthma triggers may be influenced by perceptions of how well controlled the child’s asthma is. Parental perceptions of physical activity as an asthma trigger and worse pediatric asthma control have been associated with lower levels of physical activity in children living with asthma [24]. It is possible that adjusting exercise behaviors in response to asthma control may contribute to increased weight that has been found in children living with inadequately controlled asthma [9]. Correlations have also been established between dietary intake and asthma symptoms [25]; however, there has been no research to date examining the relationship between parental feeding practices and asthma control.

This study aimed to explore parental attitudes toward child feeding, eating and exercise in children living with asthma compared with healthy controls, to better understand the higher prevalence of childhood obesity reported in children living with asthma. The objectives of the study were to:

1. Establish whether differences between parental attitudes to feeding and activity or child eating and activity levels exist in children living with and without asthma.
2. Explore any differences in the relationship between child BMI scores with parental feeding practices, parental attitudes toward physical activity, child eating behaviors and child activity levels in children living with asthma compared to healthy controls.
3. Explore whether asthma control moderates any relationships between child BMI scores with parental attitudes to feeding and activity, or child eating and activity levels.

## 2. Materials and Methods

### 2.1. Participants and Procedure

A set of standardized questionnaires were completed by parents of children living both with asthma (n = 319) and without asthma (n = 316) aged between 10 and 16 years. Participating parents were recruited through Qualtrics, an online survey company recruiting from within the UK. Qualtrics advertised the research study to a cohort who had previously been identified as parents of a child aged between 10 and 16 years, half of whom had reported that their child had asthma. Recruitment began following a favorable ethical opinion by Aston University ethics committee (project #1330). Participants provided informed consent prior to taking part in the research. 

Fourteen participants were removed from the data set because their child had comorbidities that would likely influence eating or exercise behaviors (e.g., diabetes, irritable bowel syndrome, eating disorders) or because they failed attention check questions. Data provided on parent and child weight were converted to body mass index (BMI) (kg/m^2^). To avoid inaccuracy, only participants with child height and weight data measured six months prior to data collection were included in the analysis (asthma, n = 198; no asthma, n = 194). Finally, 52 children who were classified as underweight were not included in the reported analyses as their feeding and eating behaviors are likely to differ. 

### 2.2. Measures

Data were provided for parent age, child age, ethnicity, marital status, education level, employment status and household income. Parent participants also provided height and weight data for themselves and their child. The UK 1990 reference charts were used to compute standardized BMIz scores for child gender which account for exact age and gender [26]. The following measures were also completed:

#### 2.2.1. Parental Feeding Behaviors

To measure parental feeding practices, eight subscales of the Comprehensive Feeding Practices Questionnaire (CFPQ) [27] were used: (1) child control of eating (“do you let your child eat whatever s/he wants?”), (2) use of food for emotion regulation (“when your child gets fussy, is giving him/her something to eat or drink the first thing you do? ”), (3) encouragement of energy balance and variety (“I encourage my child to try new foods”), (4) food environment (“Most of the food I keep in the house is healthy”), (5) use of food as a reward (“I offer my child his/her favorite foods in exchange for good behavior”), (6) use of restriction of food for health (“I have to be sure that my child does not eat too many sweets”), (7) restriction of food for weight control (“I encourage my child to eat less so he/she won’t get fat”) and (8) teaching about nutrition (“I discuss with my child the nutritional value of foods”). Parents responded using a Likert scale from 1 to 5. A higher mean score indicated a greater amount of the particular feeding practice. The CFPQ has been widely used and has good psychometric properties [28]; in this sample, Cronbach’s alpha scores ranged from 0.69 to 0.74, suggesting that all subscales had good internal consistency. The CFPQ has previously been validated in an adolescent sample aged 12 to 17 years of age [29].

#### 2.2.2. Parental Practices around Child Activity

The Parenting Related to Activity Measure (PRAM) [30] was used to explore parenting behaviors around child’s physical activity. Three subscales were used: (1) responsibility and monitoring (“how often are you responsible for deciding how much time your child spends engaged in physical activities?”), (2) pressure to exercise (“my child should always engage in physical activities that are available to her/him”) and (3) control of active behaviors (“I have to limit the amount of physical activity that my child engages in”). Participants’ answers were measured on a 5-point Likert scale with higher scores indicating a greater amount of the behavior or attitude. The Cronbach’s alpha scores for the responsibility/monitoring subscale, pressure to exercise subscale and control of active behaviors subscale were 0.85, 0.67 and 0.83, respectively, indicating that all subscales had good internal consistency.

#### 2.2.3. Perceptions of Child Weight

The Child Feeding Questionnaire (CFQ) [31] was used to assess parental concern about child weight (“how concerned are you about your child becoming overweight?”) and perceived child overweight (“how would you classify your child’s weight?”). Internal consistency was high with Cronbach’s alpha = 0.90. The CFQ has previously been used to examine parental feeding practices and child weight status, and within an adolescent sample [32,33].

#### 2.2.4. Child Eating

Child eating behaviors were reported by parents using four subscales from the Children’s Eating Behaviour Questionnaire (CEBQ) [34]. The CEBQ consists of 8 subscales; 4 food-approach subscales and 4 food-avoidance subscales [35]. The food-approach subscales represent behaviors associated with greater food intake and an increased obesity risk and have therefore been chosen for this study [36]. These subscales included (1) food responsiveness (“my child’s always asking for food?”), (2) emotional overeating (“my child eats more when worried?”), (3) enjoyment of food (“my child enjoys eating?”) and (4) desire to drink (“if given the chance my child would drink continuously throughout the day”). Responses were provided using a Likert scale with higher scores indicated a greater amount of the particular eating behavior. Cronbach’s alpha values ranged from 0.88 to 0.90, indicating good internal consistency. The CEBQ has previously been used with families of children who are of a healthy weight and overweight and has been validated in adolescents aged 12–16 years of age [36,37,38].

#### 2.2.5. Child Activity

The Godin Leisure-Time Exercise Questionnaire (GLTEQ) [39] was used to explore children’s physical activity levels during a typical week. The GLTEQ measures how many times children engage in periods of 15 min of strenuous (e.g., running), moderate (e.g., fast walking) and mild exercise (e.g., yoga), in an average week. To create a leisure time score, the following calculation was summed: (3 x mild) + (5 x moderate) + (9 x strenuous). A separate question enquires how often their child worked up sweat during physical activity. This is measured on a 3-point Likert scale from often to never/rarely. The Cronbach’s alpha for the leisure time score was 0.73 indicating that the scale had good internal consistency. The GLTEQ has been widely used, including with adolescents with asthma [40].

#### 2.2.6. Asthma Control

Children’s asthma control was measured using the Asthma Control Questionnaire (ACQ) [41]. Participants were asked to recall their child’s symptoms over the last week using questions such as “in general, during the past week how much of the time did you wheeze?” A 7-point Likert scale was used to record responses. Higher scores indicated poorer asthma control. A total score of >1.50 can be used to categorize inadequately controlled asthma [42], and therefore used in this study. In a population aged 6–17 years of age, the ACQ has been found to correlate with asthma rescue medication use and urgent medical care assistance [43] and has good test–retest reliability over time [44]. 

### 2.3. Statistical Analysis

Normality tests indicated that data were not normally distributed. Consequently, non-parametric tests were used in the analysis where possible. A criterion alpha of *p* < 0.05 was used to establish significance. First, Mann–Whitney U and Chi square tests were used to explore participant and child characteristics. Second, Mann–Whitney U tests were used to compare parental feeding practices, child eating behavior and physical activity variables between groups of children with and without asthma. Controlling for significant covariates, partial Spearman’s Rho correlations were used to explore whether child BMIz scores (scores standardized for child age and gender) correlated with parental feeding, child eating and physical activity variables in the 2 groups of children. As asthma control was found to correlate with child BMIz score, moderation analyses were used to explore whether asthma control moderates the relationships between child BMI with the significantly correlated variables. Moderation analyses were conducted using the PROCESS macro [45]. A power analysis was conducted using G*Power 3.1 [46] to detect power needs for the moderated regressions. For a small effect size of f2 = 0.2 in a multiple regression with 2 predictors, using an alpha level of *p* < 0.05 with a statistical power of 0.80 G*Power suggested a minimum sample size of 52. Therefore, the sample size used was sufficient.

## 3. Results

### 3.1. Demographic Information

Characteristics of the two groups (asthma and no asthma) can be found in Table 1 (parent characteristics) and Table 2 (child characteristics). Parents of children living with asthma were significantly more likely to have a higher BMI (U = 4826, *p* = 0.014) and to have a higher household income (U = 4894, *p* = 0.044). Therefore, these variables were controlled for where possible. Children living with asthma also had a significantly higher BMIz score compared with children living without asthma (U = 16,532, *p* = 0.01).

### 3.2. Differences between Parental Attitudes toward Feeding and Exercise and Child Eating and Exercise Behaviors

Parents of children living with asthma reported significantly greater use of food to regulate emotions (*p* = 0.03), restriction of diet for weight control (*p* = 0.003) and concern about child overweight (*p* = 0.003) (see Table 3). Parents of children living with asthma were also significantly more likely to monitor child activity (*p* = 0.01), pressure children to exercise (*p* = 0.006) and control active behaviors (*p* = 0.001). For child eating variables, child emotional overeating (*p* = 0.001) and desire to drink (*p* = 0.02) were reported to be significantly higher in children living with asthma. No significant differences were found between other aspects of parents feeding, child eating or exercise behaviors. 

### 3.3. Relationships between Parental Attitudes toward Feeding and Exercise and Child Eating and Exercise Behaviors with Child Weight

Spearman’s Rho partial correlations were used to explore the relationships between parental attitudes toward feeding and exercise and child eating and exercise behaviors with child BMIz scores in the two groups (see Table 4). Parent BMI and household income were controlled for in these analyses. In both asthma and no asthma groups, child BMIz score was significantly associated with greater parental restriction of food for weight control (asthma, *p* = 0.01; no asthma, *p* = 0.001), higher concerns about the child being overweight (*p* = 0.001; *p* = 0.001), greater perceived child overweight (*p* = 0.001; *p* = 0.001), greater child emotional overeating (*p* = 0.004; *p* = 0.001), greater child food responsiveness (*p* = 0.04; *p* = 0.001) and a lower child activity score (*p* = 0.01; *p* = 0.03).

For the children with asthma, child BMIz score was significantly negatively correlated with parental teaching about nutrition (*p* = 0.03). For children without asthma, child BMIz score was also significantly correlated with greater parental use of food for emotion regulation (*p* = 0.03), lower encourage balance and variety with feeding (*p* = 0.01), more parental use of food as a reward (*p* = 0.01), more restriction of food for health (*p* = 0.001), more pressure to exercise (*p* = 0.002), greater child desire to drink (*p* = 0.006) and higher child activity frequency (*p* = 0.002).

### 3.4. Asthma Control as a Moderator to the Relationship between Restriction for Weight Control and Child Activity Level with Child BMIz

In the asthma sample, 58.8% of the children had adequately controlled asthma. Mann–Whitney U tests indicated that children living with controlled asthma had a significantly lower BMIz score (mean = 1.04, SD = 1.23) than children living with inadequately controlled asthma (mean = 1.39, SD = 1.23); (U = 3874, *p* = 0.041). Therefore, moderation analyses were used to examine whether the significant associations between child BMIz with parental restriction of food for weight control, parental teaching about nutrition, child activity score, child emotional overeating and food responsiveness were moderated by asthma control [46]. 

**Figure 1:**
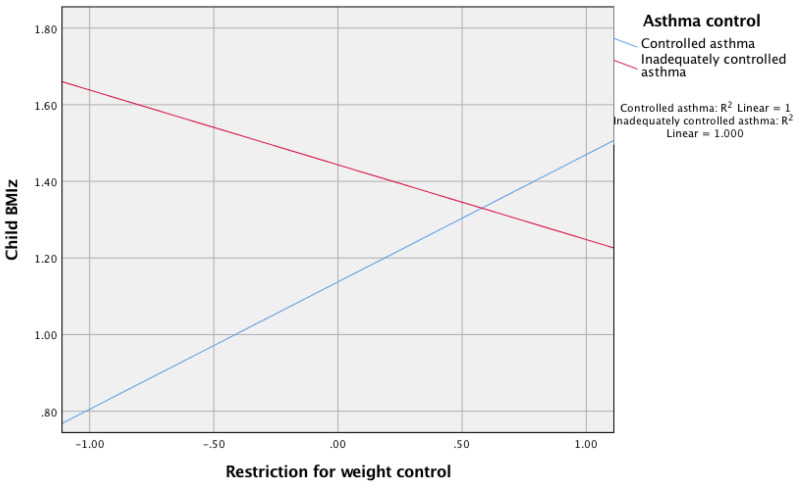
The relationship between parental restriction for weight control and child BMIz score moderated by asthma control.

Moderation analysis indicated that asthma control significantly moderated the relationship between parental restriction of food for weight control with child BMIz score; b = −0.53, 95% (Cl −0.99, −0.6), t = −2.23, *p* = 0.03. For children with adequately controlled asthma, a significant positive relationship was found between restriction for weight control and BMIz score; b = 0.33, 95% (Cl 0.05, 0.61), t = 2.36, *p* = 0.02, but when asthma was inadequately controlled, the relationship between restriction for weight control and child BMIz score was not significant; b = −0.19, 95% (Cl −0.57, 0.18), t = −1.02, *p* = 0.31 (see Figure 1).

**Figure 2:**
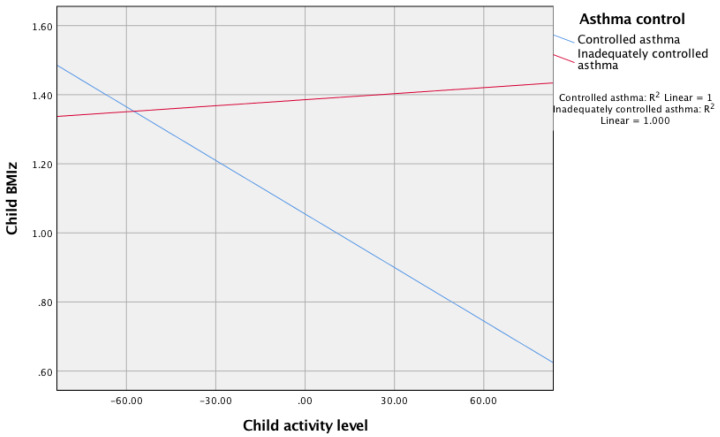
The relationship between child activity and child BMIz score moderated by asthma control.

Moderation analysis further indicated that asthma control moderated the relationship between child activity score and BMIz score; b = 0.006, 95% (Cl 0.00, 01), t = 2.13, *p* = 0.035. For children with adequately controlled asthma, there was a significant negative relationship between child activity and child BMIz score, b = −0.002, 95% (Cl −0.0094, −0.009), t = −2.42, *p* = 0.02. However, when asthma was inadequately controlled, the relationship between activity and child BMIz score was not significant, b = −0.0006, 95% (Cl −0.0027, 0.0038), t = 0.35, *p* = 0.73 (see Figure 2).

Further moderation analyses indicated that asthma control did not moderate the relationships between child BMIz score with teaching about nutrition (b = 0.15, 95% (Cl −0.47, 0.78), t = 0.48, *p* = 0.63), child emotional overeating (b = −0.28, 95% (Cl −0.64, 0.07), t = −1.54, *p* = 0.13) or child food responsiveness (b = −0.22, 95% (Cl −0.56, 0.12), t = −1.3, *p* = 0.19).

## 4. Discussion

Parents of children living with asthma reported that their children engaged in more unhealthy eating behaviors such as emotional overeating and greater desire to drink compared with children living without asthma. The children also had a significantly higher BMIz score compared with peers without asthma, and their parents reported greater concern regarding their children’s weight. Parents of children living with asthma also reported using more controlling practices when feeding their children (using food to regulate emotion, restriction of food for weight control) as well as more controlling practices in relation to exercise (e.g., increased monitoring of child activity, higher pressure on child to exercise and greater control of active behaviors). These results suggest that children living with asthma are exposed to different environmental factors that influence and shape their activity and weight, compared with their peers without asthma. 

A significantly higher BMIz score in children living with asthma supports previous findings that this group have an increased obesity risk [8,9]. Parental restriction of food for weight control was found to correlate with higher child BMIz in both groups. Parental restriction of food for weight control has previously been associated with greater weight in a general child population [47], but it has not before been reported in a pediatric asthma sample. The current study adds to existing evidence and additionally shows that this feeding practice is increased in parents of children living with asthma. Parental restriction of food may both, therefore, predict child BMIz and be used in response to parental concerns about child overweight. Indeed, in longitudinal studies, greater parental restriction of food has been shown to predict the development of child eating in response to negative emotions over time [10]. These findings support those showing that adolescents living with asthma are more likely to eat when sad compared to peers without asthma [17]. 

Interestingly, we found that teaching children about nutrition was only associated with child BMIz scores in the asthma group. Education on nutrition and its relationship with asthma outcomes may help to increase families’ understanding of the importance of maintaining a healthy diet for weight management and as a means to enhance asthma control. Previous literature has reported that diets high in sodium and fat can increase bronchial hyper-responsiveness and asthma symptoms [48]. In comparison, a Mediterranean diet high in fruits and vegetables is reported to reduce airway inflammation [49]. Previous nutritional interventions have been found to improve asthma outcomes and reduce child weight, indicating that integrating personalized nutrition plans with medical treatment could support behavior change in those living with asthma [50,51,52]. Additionally, interventions to improve children’s dietary intake could reduce children’s risk of developing further comorbidities that could complicate asthma management. Existing evidence highlights how poor early nutritional intake can impact upon child neurodevelopment and increase susceptibility to future comorbidities, such as heart disease and type 2 diabetes [53,54]. Further research on the effectiveness of such interventions in an asthma population is needed. 

Significantly higher parental reports of monitoring and control of child active behaviors also support previous research, suggesting that parents of children living with asthma worry more about their child’s exercise limitations [24]. In addition, parental attitudes toward physical activity did not correlate with child BMIz in our asthma sample. It may be that parents’ attitudes do not influence child activity levels or that participation is still encouraged but in safe, controlled environments [55]. Activity levels were found to correlate with child BMIz score in the asthma group, whereas exercise frequency did not. For children living with asthma, activity participation may be more important for weight management than strenuous exercise. This contrasts with recommendations to increase activity intensity for weight management in children living with asthma [56]. It is possible that the role regular exercise participation has in stretching in the airways, reducing the risk of airway hyper-responsiveness and increasing exercise capacity may improve asthma control and support engagement in weight management behaviors [2,57,58]. Meanwhile, there is conflicting evidence on the effect high-intensity exercise can have on asthma symptoms and inflammation [59,60], factors that may mediate future exercise engagement and weight management. 

A significant association was also found between inadequately controlled asthma and higher child BMIz, supporting previous research in this area. This study supports previous research which suggests that inadequate asthma control is associated with a higher child BMIz score [9]. Asthma control also moderated the relationship between restriction of food for weight control and child BMIz score, and between child activity levels and child BMIz score. Specifically, when asthma symptoms were controlled, parental restriction of food for weight control predicted higher child BMIz scores, and higher child activity predicted lower child BMIz scores. However, these relationships were not significant in the children with inadequately controlled asthma. These results suggest that the relationship between restriction of food for weight control and between child activity levels with child BMIz may be similar between parents of children with controlled asthma and parents of children living without asthma [11]. These relationships were not found when asthma was inadequately controlled, suggesting that there may be other factors influencing weight gain in children who have inadequately controlled asthma. However, in families with uncontrolled asthma, there may be other factors that are more significant that are influencing child weight. One explanation could be the high-dose steroid therapy that is used in difficult to control or in “therapy-resistant” asthma [61]. Han and colleagues [62] found a positive association between steroid dose and weight gain. Another possible explanation is contributor to weight could be non-adherence to asthma medication, a behavior associated with worse asthma control and increased weight [63,64]. It is also possible that parental feeding practices may vary in response to parents’ perceptions of illness control, beliefs about how to manage asthma symptoms and methods used to alleviate child anxiety [12,13,16,65,66]. These findings suggest that healthcare professionals may need to consider other factors when providing weight management support to families with uncontrolled asthma.

To our knowledge, this is the first study to explore the relationships between parental feeding and exercise practices in a sample of children living with and without asthma. While the cross-sectional nature of this study means that the directionality of the relationships found cannot be determined, these findings highlight important factors that necessitate further consideration by practitioners and researchers developing pediatric asthma weight management interventions. Nevertheless, there are limitations to this study which should be considered. The first is the use of self-reported data to analyze child weight, diet, activity and asthma control. While only participants with recent BMI data were included, it would be better to objectively measure BMI. Future studies may consider using child involvement, physiological assessment or 24-h recall diaries as more robust measures. Furthermore, although controlled for where possible in analyses, the difference in parent BMI between the asthma and no asthma group should be noted when inferences are drawn from these findings. Additionally, to develop a more comprehensive knowledge of the family environment in childhood asthma, families should also be purposefully recruited from more diverse backgrounds. This would support understanding of how family attitudes and behaviors vary, allowing for greater generalization to other populations. Nevertheless, the findings of this study suggest there is a relationship between parental feeding and parenting around child activity and asthma control that warrants further investigation.

## 5. Conclusions

We know that children living with asthma are likely to have an increased risk of living with overweight than their peers without asthma. This study shows that differences in parental attitudes toward feeding and exercise and child eating and exercise behaviors between families with and without asthma may help to explain the increased obesity risk in this population. Furthermore, in controlled asthma, restriction of food for weight management predicted a higher child BMI, and greater child activity predicted lower child BMI. These relationships were not found for children with inadequately controlled asthma, suggesting that in this population, other factors may be influencing child weight. Such findings highlight the benefit of healthcare professionals integrating exercise and dietary interventions into asthma care to support asthma and weight management; however, further examination is necessary to understand the relationships highlighted by this research.

## Figures and Tables

**Table 1:** Parent characteristics.

	Asthma (n = 310)	No Asthma (n = 311)
Parent age (mean, SD)	41.24 (8.11)	42.21 (8.21)
Parent gender (% female)	66.1	75.6
Parent BMI (mean, SD)	27.44 (8.58)	26.18 (7.03)
Parent marital status (%)		
Single	13.5	11.3
Married	61.3	54.7
Co-habiting	16.8	21.2
Divorced	6.8	8
Widowed	0.3	1.9
Other	1.3	2.9
Parent education status (%)		
To age 16	17.7	22.2
AS-Level/A-Level/Other equivalent	30.6	32.5
Apprenticeship	7.4	7.1
Bachelor’s degree	27.7	25.4
Postgraduate degree	14.2	9
Education level not specified	2.3	3.9
Parent employment status (%)		
Unemployed	5.2	2.6
Homemaker	17.4	24.1
Full time employment	52.6	40.2
Part time employment	14.5	18
Self-employed	6.1	9.6
Retired	0.6	1.3
Student	0.6	0.6
Other	2.9	3.5
Parent ethnicity (%)		
White British	87.4	85.5
Other	12.6	14.6
Household income (%)		
Less than £10,000	3.2	2.6
£10,000–£19,999	16.1	21.2
£20,000–£29,999	22.9	26
£30,000–£39,999	16.8	18.6
£40,000–£49,999	13.5	11.3
£50,000–£74,999	17.1	11.9
£75,000–£99,999	5.5	3.9
Over £100,000	2.6	1
Prefer not to say	2.3	3.5

BMI, body mass index.

**Table 2:** Child characteristics.

	Asthma	No Asthma
Child age (mean, SD)	12.89 (1.94)	12.52 (1.94)
Child gender (% female)	41.6	48.6
Child BMI z-score (mean, SD) (n = 198 vs. 194)	1.21 (1.25)	0.89 (1.18)
Child Comorbidities (%)	25.5	14.1

BMI, body mass index.

**Table 3:** Descriptive statistics and comparisons between groups of parents of children living with and without asthma on parental feeding, child eating and exercise variables and child weight.

	Children with Asthma—Mean (SD)	Children without Asthma—Mean (SD)	Mann–Whitney U Test
**Parental Feeding**			
Child Control	2.8 (0.71)	2.79 (0.68)	47,968
Emotion Regulation	2.21 (0.87)	2.02 (0.7)	43,273 *
Encourage balance and variety	4.42 (0.57)	4.42 (0.55)	48,150
Environment	3.59 (0.77)	3.65 (0.73)	45,599
Restriction for weight control	3.08 (0.79)	2.88 (0.79)	41,602 **
Food as a reward	2.63 (1.1)	2.54 (1.09)	45,766
Restriction for health	3.55 (1.02)	3.52 (0.72)	47,876
Teaching about nutrition	4.53 (0.72)	4.43 (0.81)	45,242
**Parenting Practices of Child Activity**			
Responsibility/monitoring	3.52 (0.82)	3.34 (0.83)	42,521 *
Pressure to exercise	3.73 (0.85)	3.53 (0.91)	42,131 **
Control of active behaviours	2.35 (1.09)	2.05 (1.03)	40,002 **
**Perceptions of Child Weight**			
Concerns about child overweight	2.76 (1.3)	2.45 (1.33)	41,697 **
Perceived child overweight	3.1 (0.58)	3.05 (0.48)	47,020
**Child Eating**			
Enjoyment of food	3.84 (0.77)	3.89 (8)	46,243
Emotional overeating	2.47 (1.04)	2.17 (0.82)	40,491 **
Desire to drink	2.84 (1.08)	2.63 (1.04)	42,855 *
Food responsiveness	2.81 (1.08)	2.66 (1)	44,701
**Child Activity**			
Activity Score	65.36 (61.21)	65.78 (59.25)	47,785
Activity frequency	1.75 (0.66)	1.81 (0.69)	45,909

* *p* < 0.05, ** *p* < 0.01.

**Table 4:** Spearman’s Rho partial correlations correlating child BMIz score with parental feeding practices, parenting practices around child activity, parental concerns around child weight, child eating and child activity levels.

	Child BMIz Score	
	Children with Asthma (n = 198)—R	Children without Asthma (n = 194)—R
**Parental Feeding**		
Child Control	−0.03	0.12
Emotion Regulation	0.12	0.14 *
Encourage balance and variety	−0.06	−0.16 *
Environment	−0.03	−0.10
Restriction for weight control	0.16 *	0.31 **
Food as a reward	0.08	0.16 *
Restriction for health	0.08	0.24 **
Teaching about nutrition	−0.13 *	−0.02
**Parenting Practices of Child Activity**		
Responsibility/monitoring	0.01	0.09
Pressure to exercise	0.03	0.21 **
Control of active behaviours	0.06	0.05
**Perceptions of child weight**		
Concerns about child overweight	0.24 **	0.37 **
Perceived child overweight	0.28 **	0.35 **
**Child Eating**		
Enjoyment of food	0.08	0.05
Emotional overeating	0.19 **	0.29 **
Desire to drink	0.10	0.19 **
Food responsiveness	0.13 *	0.30 **
**Child Activity**		
Activity Score	−0.16 *	−0.14 *
Activity frequency	0.09	0.21 **

* *p* < 0.05, ** *p* < 0.01.

## Data Availability

The data presented in this study are available on request from the corresponding author.

## References

[1] World Health Organization Asthma Key Facts. https://www.who.int/respiratory/asthma/en/.

[2] Global Initiative for Asthma Global Strategy for Asthma Management and Prevention. https://ginasthma.org/gina-reports/.

[3] Nunes C., Pereira A.M., Morais-Almeida M. (2017). Asthma costs and social impact. Asthma Res. Pract..

[4] Keeble E., Kosarova L. Focus on: Emergency Hospital Care for Children and Young People. QualityWatch. https://www.nuffieldtrust.org.uk/files/2018-10/1540142848_qualitywatch-emergency-hospital-care-children-and-young-people-full.pdf.

[5] Sundbom F., Malinovschi A., Lindberg E., Alving K., Janson C. (2016). Effects of poor asthma control, insomnia, anxiety and depression on quality of life in young asthmatics. J. Asthma.

[6] Baffi C.W., Winnica D.E., Holguin F. (2015). Asthma and obesity: Mechanisms and clinical implications. Asthma Res. Pract..

[7] Gross E., Lee D.S., Hotz A., Ngo K.C., Rastogi D. (2018). Impact of obesity on asthma morbidity during a hospitalization. Hosp. Pediatrics.

[8] Carpaij O.A., van den Berge M. (2018). The asthma-obesity relationship: Underlying mechanisms and treatment implications. Curr. Opin. Pulm. Med..

[9] Forno E., Celedón J.C. (2017). The effect of obesity, weight gain, and weight loss on asthma inception and control. Curr. Opin. Allergy Clin. Immunol..

[10] Sahoo K., Sahoo B., Choudhury A.K., Sofi N.Y., Kumar R., Bhadoria A.S. (2015). Childhood obesity: Causes and consequences. J. Fam. Med. Prim. Care.

[11] Farrow C.F., Haycraft E., Blissett J.M. (2015). Teaching our children when to eat: How parental feeding practices inform the development of emotional eating: A longitudinal experiment design. Am. J. Clin. Nutr..

[12] Scaglioni S., De Cosmi V., Ciappolino V., Parazzini F., Bramilla P., Agostoni C. (2018). Factors influencing children’s eating behaviours. Nutrients.

[13] Kral T.V.E., Souders M.C., Thompkins V.H., Remiker A.M., Eriksen W.T., Pinto-Martin J.A. (2015). Child eating behaviours and caregiver feeding practices in children with autism spectrum disorders. Public Health Nurs..

[14] Allen H.A., Chambers A., Blissett J., Chechlacz M., Barrett T., Higgs S., Nouwen A. (2016). Relationships between parental feeding practices and neural responses to food cues in adolescents. PLoS ONE.

[15] Castro-Rodriguez J.A., Garcia-Marcos L. (2017). What are the effects of a Mediterranean diet on allergies and asthma in children?. Front. Pediatrics.

[16] Borhani R., Asadi N., Mohsenpour M. (2012). The experiences of mothers with asthmatic children: A content analysis. J. Caring Sci..

[17] Moreau D., Kalaboka S., Choquet M., Annesi-Maesano I. (2009). Asthma, obesity, and eating behaviors according to the Diagnostic and Statistical Manual of Mental Disorders IV in a large population-based sample of adolescents. Am. J. Clin. Nutr..

[18] Groth S., Rhee H., Kitzman H. (2016). Relationships among obesity, physical activity and sedentary behaviour in young adolescents with and without lifetime asthma. J. Asthma.

[19] Bhatia R., Abu-Hasan M., Weinberger M. (2019). Exercise-induced dyspnea in children and adolescents: Differential diagnosis. Pediatrics Ann..

[20] Park S.J., Sawyer S.M., Glaun D.E. (1996). Childhood asthma complicated by anxiety: An application of cognitive behavioural therapy. J. Pediatric Child Health.

[21] Sicouri G., Sharpe L., Hudson J.L., Dudeney J., Jaffe A., Selvadurai H., Lorimer S., Hunt C. (2017). Threat interpretation and parental influences for children with asthma and anxiety. Behav. Res. Ther..

[22] Bruzzese J.M., Reigada L.C., Lamm A., Wang J., Li M., Zandieh S.O., Klein R.G. (2016). Association of youth and caregiver anxiety and asthma care among urban young adolescents. Acad. Pediatrics.

[23] Kornblit A., Cain A., Bauman L.J., Brown N.M., Reznik M. (2018). Parental perspectives of barriers to physical activity in urban schoolchildren with asthma. Acad. Pediatrics.

[24] Koinis-Mitchell D., Kopel S.J., Esteban C.A., Seifer R., Vehse N.W., Chau S., Jelalian E. (2017). Asthma status and physical activity in urban children. Am. J. Respir. Crit. Care Med..

[25] Guillenminault L., Williams E.J., Scott H.A., Berthon B.S., Jensen M., Wood L.G. (2017). Diet and asthma: Is it time to adapt our message?. Nutrients.

[26] Freeman J.V., Cole T.J., Chinn S., Jones P.R., White E.M., Preece M.A. (1995). Cross sectional stature and weight reference curves for the UK, 1990. Arch. Dis. Child..

[27] Musher-Eizenman D., Holub S. (2007). Comprehensive feeding practices questionnaire: Validation of a new measure of parental feeding practices. J. Pediatric Psychol..

[28] Mais L.A., Warkentin S., Latorre M.R.D.O., Carnell S., Taddei J.A.D.A.C. (2015). Validation of the comprehensive feeding practices questionnaire among Brazilian families of school-aged children. Front. Nutr..

[29] Fleary S.A., Ettienne R. (2019). The Relationship between food parenting practices, parental diet and their adolescents’ diet. Appetite.

[30] Haycraft E., Powell F., Meyer C. (2015). Activity-related parenting practices: Development of the parenting related to activity measure (PRAM) and links with mothers’ eating psychopathology and compulsive exercise beliefs. Eur. Eat. Disord. Rev..

[31] Birch L.L., Fisher J.O., Grimm-Thomas K., Markey C.N., Sawyer R., Johnson S.L. (2001). Confirmatory factor analysis of the child feeding questionnaire: A measure of parental attitudes, beliefs and practices about child feeding and obesity proneness. Appetite.

[32] Ek A., Sorjonen K., Eli K., Lindberg L., Nyman J., Marcus C., Nowicka P. (2016). Associations between parental concerns about pre-schoolers’ weight and eating and parental feeding practices: Results from analyses of the child eating behaviour questionnaire, the child feeding questionnaire, and the lifestyle behaviour checklist. PLoS ONE.

[33] Loth K.A., MacLehose R.F., Larson N., Berge J.M., Neumark-Sztainer D. (2016). Food availability, modelling and restriction: How are these different aspects of the family eating environment related to adolescent dietary intake?. Appetite.

[34] Wardle J., Guthrie C.A., Sanderson S., Rapoport L. (2007). Development of the children’s eating behaviour questionnaire. J. Child Psychol. Psychiatry.

[35] Quah P.L., Fries L.R., Chan M.J., Fogel A., McCrickerd K., Goh A.T., Aris I.A., Lee Y.S., Pang W.W., Basnyat I. (2019). Validation of the Children’s Eating Questionnaire in 5 and 6 year-old children: The GUSTO cohort study. Front. Psychol..

[36] Mallan K.M., Daniels L.A., Nicholson J.M. (2017). Obesogenic eating behaviours mediate the relationships between psychological problems and BMI in children. Obesity.

[37] Dos Passos D.R., Gigante D.P., Maciel F.V., Matijasevich A. (2015). Children’s eating behaviour: Comparison between normal and overweight children from a school in Pelotas, Rio Grande do Sul, Brazil. Rev. Paul. Pediatr..

[38] de Groot C.J., van den Akker E.L.T., Rings E.H.H.M., Delemarre-van de Waal H.A., van der Grond J. (2016). Brain structure, executive function and appetitive traits in adolescent obesity. Pediatric Obes..

[39] Godin G., Shepard R.J. (1985). A simple method to assess exercise behaviour in the community. Can. J. Appl. Sport Sci..

[40] Suorsa K.I., Cushing C.C., Mullins A.J., Meier E., Tackett A.P., Junghans A., Chaney J.M., Mullins L.L. (2016). Adolescents and young adults with asthma and allergies: Physical activity, self-efficacy, social support, and subsequent psychosocial outcomes. Child. Health Care.

[41] Juniper E.F., O’Bryne P.M., Guyatt G.H., Ferrie P.J., King D.R. (2001). Development and validation of a questionnaire to measure asthma control. Eur. Respir. J..

[42] Olaguibel J.M., Quirce S., Juliá B., Fernández C., Fortuna A.M., Molina J., Plaza V., MAGIC Study Group (2012). Measurement of asthma control according to global initiative for asthma guidelines: A comparison with the asthma control questionnaire. Respir. Res..

[43] Nguyen J.M., Holbrook J.T., Wei C.Y., Gerald L.B., Teague W.G., Wise R.A. (2014). Validation and psychometric properties of the Asthma Control Questionnaire among Children. J. Allergy Clin. Immunol..

[44] Juniper E.F., Gruffyd-Jones K., Ward S., Svensson K. (2010). Asthma control questionnaire in children: Validation, measurement properties, interpretation. Eur. Respir. J..

[45] Hayes A.F. (2017). Introduction to Mediation, Moderation, and Conditional Process Analysis.

[46] Faul F., Erdfelder S., Buchner A., Lang A.G. (2009). Statistical power analyses using G*Power 3.1: Tests for correlation and regression analyses. Behav. Res. Methods.

[47] Freitas F.R., Moraes D.E.B., Warkentin S., Mais L.A., Ivers J.F., Taddei J.A.A.C. (2019). Maternal restrictive feeding practices for child weight control and associated characteristics. J. Pediatr..

[48] Wood L.G. (2017). Diet, obesity, and asthma. Ann. Am. Thorac..

[49] Douros K., Thanopoulou M.I., Boutopoulou B., Papadopoulou A., Papadimitriou A., Fretzayas A., Priftis K.N. (2019). Adherence to the Mediterranean diet and inflammatory markers in children with asthma. Allergol. Immunopathol..

[50] Jensen M.E., Gibson P.G., Collins C.E., Hilton J.M., Wood L.G. (2013). Diet-induced weight loss in obese children with asthma: A randomized controlled trial. Clin. Exp. Allergy.

[51] Luna-Pech J.A., Torres-Mendoza B.M., Luna-Pech J.A., Garcia-Cobas C.Y., Navarrete-Navarro S., Elizalde-Lozano A.M. (2014). Normacarloric diet improves asthma related quality of life in obese pubertal adolescents. Int. Arch. Allergy Immunol..

[52] Van Leeuwen J.C., Hoogstrate M., Duiverman E.J., Thio B.J. (2014). Effects of dietary induced weight loss on exercise-induced bronchoconstriction in overweight and obese children. Pediatric Pulmonol..

[53] Bhutta Z.A., Guerrant R.L. (2017). Neurodevelopment, nutrition, and inflammation: The evolving global child health landscape. Pediatrics.

[54] Prescott S.L., Fewtrell M.S., Haschke F., Prescott S.L. (2016). Early nutrition as a major determinant of ‘immune health’: Implications for allergy, obesity and other noncommunicable diseases. Preventive Aspects of Early Nutrition.

[55] Shaw M.R., Katz J., Benavides-Vaello S., Oneal G., Holliday C. (2017). Views on exercise: A grounded theory exploration of the creation of exercise perceptions in Hispanic children with asthma. Hisp. Health Care Int..

[56] Moonie S., Hogan M.B. (2018). Challenges for the clinician: Physical activity among severe asthmatic patients with comorbid obesity. J. Allergy Clin. Immunol..

[57] Lang J.E., Hossain J., Smith K., Lima J.J. (2012). Asthma severity, exacerbation risk, and controller treatment burden in underweight and obese children. J. Asthma.

[58] Fredberg J.J. (1998). Airway smooth muscle in asthma: Flirting with disaster. Eur. Respir. J..

[59] Lövström L., Emtner M., Alving K., Nordall L., Borres M.P., Janson C., Malinovschi A. (2015). High levels of physical activity are associated with poorer control asthma in young females but not males. Respirology.

[60] Toennensen L.L., Meteran H., Hostrup M., Geiker N.R.W., Jensen C.B., Porsbjerg C., Astrup A., Bangsbo J., Parker D., Backer V. (2018). Effects of exercise and diet in nonobese asthma patients—A randomized controlled trial. J. Allergy Clin. Immunol. Pract..

[61] Sly P.D., Holt P.G. (2019). Predicting steroid responsiveness in asthmatic children: Are we there yet?. J. Allergy Clin. Immunol..

[62] Han J., Nguyen J., Kim Y., Geng B., Romanowski G., Alejandro L., Proudfoot J., Xu R., Leibel S. (2019). Effect of inhaled corticosteroid use on weight (BMI) in pediatric patients with moderate-severe asthma. J. Asthma.

[63] Klok T., Kaptein A.A., Brand P.L.P. (2015). Non-adherence in children with asthma reviewed: The need for improvement of asthma care and medical education. Pediatric Allergy Immunol..

[64] Longo C., Bartlett G., Schuster T., Ducharme F.M., MacGibbon B., Barnett T.A. (2019). Weight status and nonadherence to asthma maintenance therapy among children enrolled in a public drug insurance plan. J. Asthma.

[65] Selby L., Beresford F., Saglani S. (2018). Emotional distress in children with problematic severe asthma is associated with parental anxiety and depression. Eur. Respir. J..

[66] Steinsbekk S., Barker E.D., Llewellyn C., Fildes A. (2017). Emotional feeding and emotional eating: Reciprocal processes and the influence of negative affectivity. Child Dev..

